# Preliminary Epidemiology of Human Infections with Highly Pathogenic Avian Influenza A(H7N9) Virus, China, 2017

**DOI:** 10.3201/eid2308.170640

**Published:** 2017-08

**Authors:** Lei Zhou, Yi Tan, Min Kang, Fuqiang Liu, Ruiqi Ren, Yali Wang, Tao Chen, Yiping Yang, Chao Li, Jie Wu, Hengjiao Zhang, Dan Li, Carolyn M. Greene, Suizan Zhou, A. Danielle Iuliano, Fiona Havers, Daxin Ni, Dayan Wang, Zijian Feng, Timothy M. Uyeki, Qun Li

**Affiliations:** Authr affiliations: Chinese Center for Disease Control and Prevention, Beijing, China (L. Zhou, R. Ren, Y. Wang, T. Chen, C. Li, D. Li, D. Ni, D. Wang, Z. Feng, Q. Li);; Guangxi Center for Disease Control and Prevention, Nanning, China (Y. Tan, Y. Yang);; Guangdong Center for Disease Control and Prevention, Guangzhou, China (M. Kang, J. Wu);; Hunan Center for Disease Control and Prevention, Changsha, China (F. Liu, H. Zhang);; Centers for Disease Control and Prevention, Atlanta, Georgia, USA (C.M. Greene, S. Zhou, A.D. Iuliano, F. Havers, T.M. Uyeki)

**Keywords:** highly pathogenic avian influenza, H7N9, HPAI, LPAI, epidemiologic characteristics, influenza, infectious diseases, viruses, China, respiratory infections, epidemiology, zoonoses

## Abstract

We compared the characteristics of cases of highly pathogenic avian influenza (HPAI) and low pathogenic avian influenza (LPAI) A(H7N9) virus infections in China. HPAI A(H7N9) case-patients were more likely to have had exposure to sick and dead poultry in rural areas and were hospitalized earlier than were LPAI A(H7N9) case-patients.

Since the first human infections with avian influenza A(H7N9) virus were identified in early 2013 (*1*), mainland China has experienced 5 epidemics of human infections with A(H7N9) virus (*2*). As of March 31, 2017, a total of 1,336 cases of laboratory-confirmed A(H7N9) virus infections were detected; case-fatality proportion was ≈40%. The fifth epidemic began September 1, 2016, and the number of A(H7N9) virus infection cases has surged since December 2016 (*2*). Until recently, all human infections were with low pathogenic avian influenza (LPAI) A(H7N9) virus, which causes little or no disease in infected poultry. Risk factors for human infection with LPAI A(H7N9) virus include visiting a live poultry market (LPM) or raising backyard poultry, and mortality is higher among older adults with chronic comorbid conditions (*3*,*4*).

On February 18, 2017, the National Health and Family Planning Commission of China reported genetic sequencing results on virus isolates from 2 patients from Guangdong Province who had A(H7N9) virus infection (initially reported to the Chinese Center for Disease Control and Prevention [China CDC] in January 2017) that were consistent with highly pathogenic avian influenza (HPAI) viruses. Insertions at the hemagglutinin gene cleavage site consistent with HPAI A(H7N9) virus were confirmed by the Chinese National Influenza Center (CNIC) (*5*). Detection of HPAI A(H7N9) virus in LPMs in Guangdong Province was reported on February 20, 2017 (*6*). An additional case of HPAI A(H7N9) virus infection was identified in Taiwan in a patient with illness onset in Guangdong Province (*5*,*7*,*8*). To assess whether disease severity in humans has changed with HPAI A(H7N9) compared with LPAI A(H7N9) virus infection, we described the epidemiologic characteristics of cases of HPAI and LPAI A(H7N9) virus infections identified during the current fifth epidemic in mainland China.

## The Study

Detection, reporting, and confirmation of HPAI A(H7N9) virus infection was the same as for LPAI A(H7N9) and HPAI A(H5N1) virus infections, as previously described (*3*,*9*,*10*). Since the first case of HPAI A(H7N9) virus infection was identified in 2017, genetic analyses are performed at provincial China CDC laboratories or at CNIC on respiratory specimens collected from all case-patients identified with A(H7N9) virus infection to distinguish between HPAI and LPAI A(H7N9) viruses. Field investigations and data collection protocols for HPAI A(H7N9) cases were the same as for LPAI A(H7N9) cases (*3*).

We extracted information from field investigation reports and the notifiable infectious surveillance system to describe the demographic, clinical, and epidemiologic characteristics of HPAI A(H7N9) case-patients. We used descriptive statistics to compare HPAI A(H7N9) cases with all LPAI A(H7N9) cases reported throughout mainland China and with LPAI A(H7N9) cases identified in the same provinces as HPAI A(H7N9) cases during the fifth epidemic reported as of March 31, 2017. Collection and analyses of data from human infections with A(H7N9) virus were determined to be part of an ongoing public health investigation of emerging outbreaks and thus were exempt from institutional review board assessment in China (*3*).

**Figure 1:**
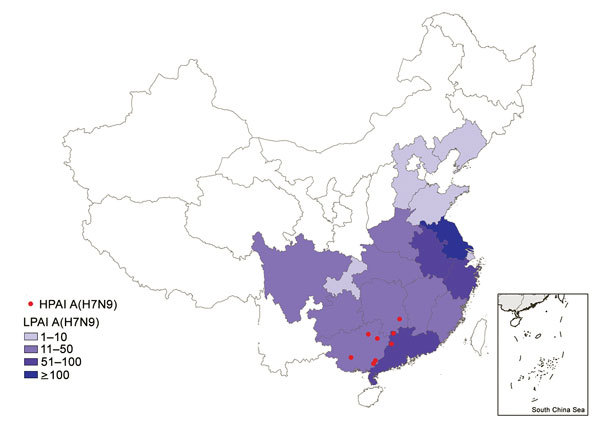
Geographic distribution of human cases of infection with HPAI A(H7N9) virus, China, September 1, 2016–March 31, 2017. The red circles indicate the counties with HPAI A(H7N9) virus infections within Guangxi, Guangdong, and Hunan provinces during the fifth epidemic. Shading indicates the total numbers of LPAI A(H7N9) virus infections by province during the fifth epidemic. HPAI, highly pathogenic avian influenza; LPAI, low pathogenic avian influenza.

**Figure 2:**
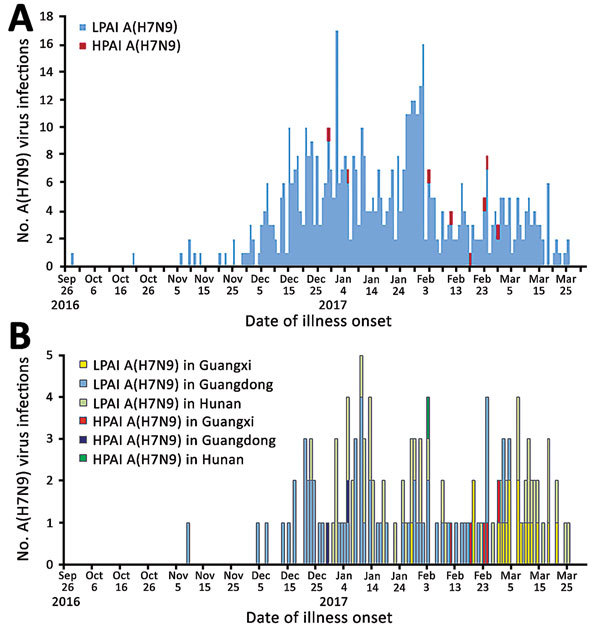
Human infections with HPAI or LPAI A(H7N9) viruses, by illness onset date, China, September 1, 2016–March 31, 2017. A) Dates of illness onset for the 8 HPAI A(H7N9) cases compared with those for all LPAI A(H7N9) cases. B) Dates of illness onset for the 8 HPAI A(H7N9) cases compared with those for LPAI A(H7N9) cases in 3 provinces (Guangxi, Guangdong, and Hunan).

Eight cases of HPAI A(H7N9) virus infection were identified from 3 provinces in southern China (Figure 1). The first 2 case-patients had illness onset on December 30, 2016, and January 5, 2017, in Guangdong Province. Additional case-patients were identified in Hunan and Guangxi Provinces with illness onset during February 2017 (Figure 2). Of the 8 total case-patients, the median age was 57 years (range 28–71 years), and 4 (50%) were male. Most (75%) case-patients lived in rural areas, as defined previously (*4*), and all were exposed to poultry within 10 days of illness onset. Five case-patients had exposure to backyard poultry, including 4 exposed to sick or dead poultry; 2 had household exposure to poultry purchased from LPMs, including 1 with poultry that were sick and died in the home; and 1 was a poultry worker who sold and slaughtered poultry at an LPM. One cluster of HPAI A(H7N9) cases was identified in 2 adult sisters; 1 sister had household exposure to sick and dead poultry, and the other sister had exposures to sick and dead poultry at her sister’s house, to poultry brought inside her home from her sister’s house, and to her ill sister while that sister was hospitalized.

All 8 HPAI A(H7N9) case-patients were admitted to hospital a median of 2.5 days (range 0–5 days) after illness onset. All 8 case-patients received oseltamivir treatment a median of 4 days (range 1–8 days) after illness onset; 7 were admitted to an intensive-care unit, and 6 were placed on mechanical ventilation for a median of 5.5 days (range 4–7 days) after illness onset. Four case-patients died a median of 6.5 days (range 5–44 days) after illness onset, and 4 recovered and were discharged home after a median of 29 days (range 21–52 days) (Table).

**Table:** Selected characteristics of case-patients with HPAI and LPAI A(H7N9) virus infections, mainland China, September 1, 2016–March 31, 2017*

Characteristics	Infection type		p value
	HPAI, n = 8	LPAI, n = 553	
Median age (range), y	56.5 (28–71)	57 (3–91)	0.632†
Age group, y			
0–14	0	5/553 (1)	NA
15–59	5 (63)	313/553 (57)	–
>60	3 (38)	235/553 (42)	–
Sex			
M	4 (50)	400/553 (72)	0.317
F	4 (50)	153/553 (28)	–
Residence area‡			
Urban	1 (13)	193/364 (53)	0.031§
Rural	7 (88)	171/364 (47)	–
Having >1 underlying medical conditions¶	5 (63)	234/432 (54)	0.733#
Poultry exposure within 10 d of illness onset			
Any exposure to poultry	8 (100)	442/500 (90)	1.000§
Visited live poultry market	3 (38)	324/442 (73)	NA
Exposure to backyard poultry	4 (50)	98/442 (22)	NA
Occupational exposure to poultry	1 (13)	20/442 (5)	NA
Exposure to sick or dead poultry	4 (50)	43/268 (16)	0.037#
Clinical management			
Hospitalization	8 (100)	478/480 (99)	NA
Antiviral treatment	8 (100)	392/404 (97)	NA
ICU	7 (88)	323/403 (80)	1.000
Mechanical ventilation	6 (75)	221/386 (57)	0.476
Timeline of clinical management (median), d†			
Illness onset to first medical service seeking	0.5 (0–5)	2 (0–34)	0.096
Illness onset to hospitalization	2.5 (0–5)	5 (0–35)	**0.032**
Illness onset to antiviral treatment	4 (1–8)	6 (0–29)	0.168
Illness onset to diagnosis	6.5 (4–9)	8 (0–31)	0.241
Illness onset to death	6.5 (5–44)	13 (2–62)	0.180
Outcome			
Death	4 (50)	203/376 (54)	1.000
Recovered and discharged	4 (50)	173/376 (46)	–


*Values are no. (%) unless otherwise indicated. The χ^2^ test was used to compare the variables between HPAI and LPAI groups. Data were missing for some variables, and data on final outcomes were missing for case-patients with LPAI A(H7N9) virus infection who remained hospitalized as of March 31, 2017. We were not able to perform the statistical analyses to assess differences for some variables because the number of cells with expected frequency of <5 was >20% and some cells had expected frequency of <1. HPAI, highly pathogenic avian influenza; ICU, intensive-care unit; LPAI, low pathogenic avian influenza; NA, not available. †The z-test was used to compare median age and median days of the timeline of clinical management between HPAI and LPAI groups. ‡Urban was defined as cities, towns, and suburbs; rural was defined as villages and countryside (*3*). §Residence area and “any exposure to poultry” were compared between HPAI and LPAI groups by using the Fisher exact test. ¶Three HPAI case-patients had chronic cardiovascular disease, and 2 HPAI case-patients had chronic metabolic disease. #“Having >1 underlying medical conditions” and “exposure to sick or dead poultry” were compared between HPAI and LPAI groups by using the χ^2^ test for continuous correction.

Compared with all LPAI A(H7N9) case-patients reported during the fifth epidemic, HPAI A(H7N9) case-patients were significantly more likely to live in rural areas (88% vs. 47%; p = 0.031), have exposure to sick or dead poultry (50% vs. 16%; p = 0.037), and be hospitalized earlier (median 2.5 vs. 5 days; p = 0.032) (Table). No significant differences were observed in median age, sex, prevalence of underlying chronic medical conditions, median time from illness onset to starting antiviral treatment, or proportion of patients who received oseltamivir treatment, intensive-care unit admission, or mechanical ventilation (Table). Although the median time from illness onset to death (6.5 vs. 13 days) was shorter and the overall case-fatality proportion (50% vs. 37%) was higher for HPAI A(H7N9) case-patients than for LPAI A(H7N9) case-patients, these differences were not statistically significant (Table). When the analysis was restricted to the 3 provinces with HPAI A(H7N9) cases identified during the fifth epidemic, the only significant difference was a shorter median time from illness onset to death for HPAI A(H7N9) case-patients compared with LPAI A(H7N9) case-patients in Guangxi Province (5 vs. 17 days; p = 0.0192).

## Conclusions

Our preliminary findings indicate that HPAI A(H7N9) virus infection was associated with exposure to sick and dead backyard poultry in rural areas. In the ongoing fifth epidemic in mainland China, HPAI A(H7N9) case-patients were hospitalized earlier than LPAI A(H7N9) case-patients but otherwise had similar epidemiologic characteristics and disease severity.

The small number of HPAI A(H7N9) cases limited our statistical power to detect differences in epidemiologic characteristics and disease severity between HPAI and LPAI A(H7N9) case-patients. Data were not available for all variables analyzed, including outcomes for some LPAI A(H7N9) case-patients who remained hospitalized. Our findings might suggest more rapid progression and greater disease severity for HPAI A(H7N9) case-patients, because mortality was higher and the intervals from illness onset to diagnosis and to death were shorter compared with LPAI A(H7N9) case-patients; however, these differences were not significant.

Because A(H7N9) virus circulation among poultry is ongoing in mainland China, extensive efforts are needed to prevent and control the spread of LPAI and HPAI A(H7N9) viruses among poultry, including in rural areas. Avoidance of sick or dead poultry that might be infected with HPAI A(H7N9) virus can reduce transmission of HPAI A(H7N9) virus to humans. Enhanced surveillance of HPAI and LPAI A(H7N9) viruses in poultry and humans, timely virus characterization, and ongoing assessments of the epidemiology of human infections with A(H7N9) viruses are critical to guide prevention and control efforts and to provide information on the risk of these novel influenza A viruses to public health.
